# Alcohol’s Effect on Lactation

**Published:** 2001

**Authors:** Julie Mennella

**Affiliations:** Julie Mennella, Ph.D., is a member of the Monell Chemical Senses Center, Philadelphia, Pennsylvania

**Keywords:** lactation, physiological AODE (alcohol or other drug effects), breast milk, pregnancy hormones, infant, sleep disorder, developmental delay, motor coordination, alcohol-related neurodevelopmental disorder, learning

## Abstract

Although pregnant women are discouraged from drinking alcohol because of alcohol’s detrimental effect on fetal development, the lore of many cultures encourages lactating women to drink alcohol to optimize breast milk production and infant nutrition. In contrast to this folklore, however, studies demonstrate that maternal alcohol consumption may slightly reduce milk production. Furthermore, some of the alcohol consumed by a lactating woman is transferred to her milk and thus consumed by the infant. This alcohol consumption may adversely affect the infant’s sleep and gross motor development and influence early learning about alcohol. Based on this science, it would seem that the recommendation for a nursing mother to drink a glass of beer or wine shortly before nursing may actually be counterproductive.

Throughout most of human evolution, infants for several years after birth received their nutrients primarily from their mothers in the form of breast milk. Breast milk is a complex fluid produced by the mother’s body that fulfills a similar nutritional function as does the placenta during pregnancy. That is, it protects the infant from disease and influences certain aspects of the infant’s behavior and physiology. In essence, without successful breastfeeding, the human species would not have survived.

In many cultures a centuries-old belief persists that the process of breast-milk production and breastfeeding (i.e., lactation) can be optimized by having lactating women drink alcohol (Mennella 1999). For example, the consumption of small quantities of alcohol shortly before nursing is believed to increase milk yield, facilitate the release of the milk from the mammary glands where it is produced (i.e., the let-down), and relax both the mother and infant. In fact, this folklore was so well ingrained in American tradition that, in 1895, a major U.S. brewery produced *Malt Nutrine*, a low-alcoholic beer composed of barley malt and hops. This product was sold exclusively in drugstores and prescribed by physicians as a tonic for pregnant and lactating women and a nutritional beverage for children (Krebs 1953). Its production was halted during Prohibition because it contained more than 0.5 percent alcohol.

Even in modern times, alcohol continues to be hailed as an agent that promotes lactation (i.e., a galactagogue). For example, women in Mexico are encouraged to drink as much as two liters (i.e., one-half gallon) of *pulque*—a low-alcohol beverage made from the fermented juice of the plant *Agave atrovirens*—daily during both pregnancy and lactation. Similarly, Indochinese women in California drink wine steeped with herbs, and in Germany malt beer is considered a “magic elixir.”

Alcohol consumption among lactating women also is common in the United States. Epidemiological studies found that although lactating women were less likely to report occasional binges of heavy drinking, the “regular” drinking patterns at 1 and 3 months after giving birth (i.e., postpartum) did not differ significantly between women who elected to breastfeed and women who never breastfed (Little et al. 1990). In contrast, breast-feeding women limited their use of other drugs (e.g., were less likely to smoke cigarettes or marijuana or to use cocaine). In the same survey approximately 10 percent of lactating women reported consuming at least one drink daily. Whether these women were drinking in response to the folklore mentioned above is not known. A recent study has indicated, however, that lactating women who were either encouraged to drink or received no advice at all about alcohol reported drinking significantly more than did women who were advised not to drink (Mennella 1997).

The claims that alcohol benefits lactation are not accompanied by any controlled scientific evidence, and little research has been conducted in this area. This article reviews the existing scientific literature on alcohol’s effects on lactation. After a brief overview of the initiation and maintenance of lactation, the article describes the transfer of alcohol to human milk and the effects that maternal alcohol consumption have on the interaction between mother and infant. This discussion includes effects on milk production and milk properties (e.g., flavor), the infant’s milk intake, and the infant’s motor development and early learning.

## Overview of Lactation

Breast milk is produced by mammary glands located in the breast tissue. These glands are present from birth, but become fully functional for milk production only during pregnancy. Several hormones regulate the development of the mammary glands as well as the initiation and maintenance of lactation. The most important of these hormones are prolactin and oxytocin, both of which are produced in the pituitary gland in the brain. Prolactin, together with other hormones (e.g., estrogen and progesterone), regulates the final development of the mammary glands during pregnancy. After birth (i.e., parturition), the woman’s hormonal environment changes, and in this setting prolactin can initiate milk secretion from the mammary glands.

In addition to its role in mammary gland development and initiation of lactation, prolactin also is essential for the maintenance of lactation. During each feeding session, the infant’s suckling at the breast induces prolactin release from the pituitary gland. This prolactin release stimulates the mammary glands to produce new milk before the next feeding. The extent of prolactin release (and, consequently, the amount of milk produced) is determined by the intensity of the suckling. Thus, if an infant is hungry and nurses strongly, the resulting high levels of prolactin released from the pituitary gland ensure sufficient milk production to meet the infant’s needs. Conversely, any conditions that interfere with effective suckling will result in lower levels of prolactin release, thereby compromising milk production.

Oxytocin plays a key role in the milk let-down during nursing. Its release from the pituitary gland in response to suckling or other stimuli causes certain cells around the mammary glands to contract, thereby expelling the milk from the glands into small ducts leading to the nipple. Without this let-down reflex, the infant cannot nurse and empty the breast effectively.

## Transfer of Alcohol Into the Milk

When a lactating woman consumes alcohol, some of that alcohol is transferred into the milk. In general, less than 2 percent of the alcohol dose consumed by the mother reaches her milk and blood. Alcohol is not stored in breast milk, however, but its level parallels that found in the maternal blood. That means that as long as the mother has substantial blood alcohol levels, the milk also will contain alcohol. Accordingly, the common practice of pumping the breasts and then discarding the milk immediately after drinking alcohol does not hasten the disappearance of alcohol from the milk as the newly produced milk still will contain alcohol as long as the mother has measurable blood alcohol levels. Peak alcohol levels both in the mother’s blood and in the milk occur approximately one-half hour to an hour after drinking and decrease thereafter, although there are considerable individual differences in the timing of peak levels and in alcohol elimination rates in both milk and blood (Lawton 1985; Mennella and Beauchamp 1991). Therefore, lactating women should not nurse for several hours after drinking until their blood alcohol levels have declined again.

The question of whether exposure to alcohol in the mother’s milk can affect an infant in the short or long term has generated much speculation in the medical community. Because alcohol is excreted only to a limited extent in breast milk, many clinicians consider occasional exposure insignificant except in rare cases of intoxication in which the mother of a breast-feeding infant drinks heavily or in which a child is inadvertently fed large amounts of alcohol in a bottle. Contrary to this perception, however, the limited research that exists to date suggests that alcohol administration through the breast milk may affect the infant in several ways, such as altering milk intake and influencing infant behavior and early development and learning. These effects are discussed in the following sections.

## Alcohol’s Effect on the Breast-Feeding Process and the Infant

As mentioned earlier, folklore suggests that alcohol consumption by a lactating woman improves milk production and, in turn, the nutrition of her infant. Contrary to this assumption, however, studies have found that breast-fed infants consumed, on average, 20 percent *less* breast milk during the 3 to 4 hours following their mothers’ consumption of an alcoholic beverage (Mennella and Beauchamp 1991, 1993). This finding is consistent with the results of similar studies conducted in rats (Subramanian and Abel 1988; Swiatek et al. 1986; Vilaró et al. 1987). The observed decrease in milk intake did not occur because the infants nursed for shorter periods of time (Mennella and Beauchamp 1991, 1993) or rejected the mother’s milk because of an altered flavor following maternal alcohol consumption (Mennella 1997). Rather, maternal alcohol consumption reduced the amount of milk produced (i.e., quantity) without altering its quality (e.g., caloric content) (Mennella 1999).

As described earlier, the production and ejection of milk from the mammary gland are the result of highly synchronized hormonal processes that are governed, at least in part, by the frequency and intensity of the infant’s suckling. These hormonal processes may be influenced by alcohol consumption. For example, studies in lactating rats demonstrated that although acute alcohol administration did not affect baseline prolactin levels, it significantly inhibited suckling-induced prolactin and oxytocin release as well as milk production and, consequently, the pups’ milk intake (Subramanian and Abel 1988; Subramanian 1999). Whether acute alcohol consumption has similar effects on the hormonal milieu in lactating women is not known, however. Nor do researchers know whether chronic drinking affects the quantity and quality of milk produced in humans (see Heil et al. 1999).

Although infants consumed less milk when their mothers had consumed an alcoholic beverage compared with a nonalcoholic beverage, the mothers were apparently unaware of this difference (Mennella and Beauchamp 1993). That is, mothers who had consumed an alcoholic beverage believed their infants had ingested enough milk, reported that they experienced the sensation of milk let-down, and felt they had milk remaining in their breasts at the end of the majority of feedings. Because milk intake and the rate of milk production varies from feeding to feeding, a small difference in the infant’s milk intake may be difficult for women to perceive. With breast-fed infants, the amount of milk ingested often varies, and milk usually can be expressed from the mother’s breasts after a feeding. Perhaps one reason why the folklore that alcohol is a galactagogue has persisted for centuries is that a lactating mother does not have an immediate means of assessing whether her infant consumes more or less milk in the short term.

### Effect on Infant Sleep

Another presumed effect of maternal alcohol consumption is to relax the infant and thus promote the infant’s sleep. Studies found, however, that in the short term, acute exposure to alcohol in mothers’ milk altered the infants’ sleep-wake patterning in ways that are contrary to this medical lore (Mennella and Gerrish 1998). Infants whose mothers were light drinkers during both pregnancy and lactation slept for significantly shorter periods of time during the 3.5 hours after nursing when the mothers had consumed an alcoholic beverage than when they had consumed a nonalcoholic beverage. This reduction was due in part to a decrease in the amount of time the infants spent in active sleep.^1^ This finding is consistent with the results of studies assessing alcohol’s effect on sleep in the near-term fetus (Mulder et al. 1998), normal adults (Williams et al. 1983), and other animals (Mendelson and Hill 1978).

### Effects on Infant Development

Researchers examined the longer-term effects of alcohol consumption by lactating women in an epidemiological study of 400 breast-fed infants and their mothers. The study assessed the relationship between the mothers’ alcohol use during lactation and their infants’ development at 1 year of age (Little et al. 1989). The study found that gross motor development was slightly, but significantly, altered in infants who were exposed regularly (i.e., at least daily) to alcohol in their mothers’ milk. No significant correlation existed, however, between maternal drinking and the infants’ mental development. Furthermore, the motor and mental development of infants whose mothers drank less than one drink per day did not differ significantly from the development of infants whose mothers did not drink at all or who were formula fed.

The association between maternal drinking and delayed motor development persisted even after the investigators controlled for more than 100 potentially confounding variables, such as maternal tobacco, marijuana, and heavy caffeine use during pregnancy and the first 3 months after delivery. Moreover, the deficits in motor functioning were not attributable to alcohol-related differences in maternal behavior, because infants of heavy drinkers who were weaned at an early age had significantly higher scores on motor development than did infants of heavy drinkers who were weaned at an older age and thus were exposed to alcohol longer (Little 1990).

To explain the effects of alcohol consumed through breast milk on infant development, researchers have formulated several hypotheses (see Little et al. 1989). For example, some have suggested that the developing brain is highly sensitive even to small quantities of alcohol. Others have posited that alcohol may accumulate in the infant following repeated exposure because infants may break down (i.e., metabolize) or excrete alcohol more slowly than do adults. Some evidence suggests that infants have a limited capacity to metabolize alcohol, which in turn may render the alcohol dose more potent. For example, studies found that like alcohol, caffeine is excreted to a limited extent in breast milk and the dose presented to the infants is generally less than 2 percent of the maternal dose. Breast-fed infants are at greater risk for accumulating caffeine, however, than are older children and adults. This accumulation may be due to a lower activity in infants of an enzyme system in the liver called the cytochrome P-450 system, which is involved in caffeine breakdown. Because the same enzyme system is involved in alcohol metabolism, its reduced activity in infants could result in alcohol accumulation.

### Effects on Early Learning

In addition to the effects of maternal alcohol consumption on infant nutrition and development, experience with the sensory qualities of alcohol in the mother’s milk may affect the infant in other important ways. Animal studies have revealed that young animals (including presumably humans) form memories based on orosensory experiences during nursing and retain these memories for a considerable time (Molina et al. 1999). This observation is especially relevant because infants can detect the flavor of alcohol in mothers’ milk (Mennella 1997). Moreover, the context in which the infant experiences alcohol—that is, with the mother and during breastfeeding—consists of numerous elements that reinforce early learning, such as tactile stimulation, warmth, milk, and the mother’s voice.

Studies have demonstrated that such experiences can influence the infants’ responses to alcohol. For example, breast-fed infants differentially responded to toys that were identical in appearance but differed in scent (Mennella and Beauchamp 1998). The investigators observed infants who had been exposed to alcohol to various degrees, as inferred from questionnaires about maternal and paternal risk for alcoholism and alcohol intake, with respect to four behaviors (i.e., mouthing, looking, manipulating the toy, and vocalizing) in response to an alcohol-scented, vanilla-scented, or unscented toy. The study found infants who had more exposure to alcohol behaved differently in the presence of an alcohol-scented toy than did infants with less alcohol exposure. Specifically, infants who had more exposure to alcohol demonstrated more mouthing of the alcohol-scented toy, but not of the other toys, than did infants with less alcohol exposure. This finding is consistent with animal studies indicating that rat pups exposed to the flavor of alcohol in milk increased their mouthing rates in response to alcohol odor and were more willing to ingest alcohol-flavored solutions (Hunt et al. 1993). These results suggest that at least some of the early learning about alcohol is based on sensory experiences and is anchored to experiences with the parents.

Research on children ages 3 to 6 years also revealed that the emotional context in which parents experience alcohol, as well as their frequency of drinking, is related to children’s liking the odor of alcohol (Mennella and Garcia 2000). Children of a parent or parents who drank alcohol to “escape” problems were more likely to judge the odor of beer as unpleasant than were similarly aged children whose parents did not drink to escape. These findings are consistent with animal studies demonstrating that pups exposed to an intoxicated mother followed by pairings of alcohol odor and an arousing texture (i.e., sandpaper) later demonstrated an aversion to the texture (Molina et al. 2000). Moreover, they concur with previous reports that elementary school-aged children of alcoholic parents were more likely to report negative expectations regarding alcohol’s effects than were control children (Miller et al. 1990; Wiers et al. 1998). Thus, together with the results of Noll and colleagues (1990), the studies by Mennella and colleagues (Mennella and Garcia 2000; Mennella and Beauchamp 1998) indicate that the child’s learning about alcohol may be occurring at even younger ages than previously thought.

## Conclusions

Because of the paucity of scientific investigations on alcohol’s effects on breast-feeding, women, and consequently their infants, have had to rely on a rich folklore that has been passed down for generations. This lore relates that alcohol has galactogenic properties that facilitate milk let-down and rectify milk insufficiency as well as sedative properties that alleviate and calm the “fussy” infant. The scientific study of alcohol’s effect on the lactation process has called these assumptions into serious question, however. For example, such studies indicated that infants actually ingest less milk at the breast during the hours immediately following maternal alcohol consumption and that this diminished intake results, at least in part, from alcohol’s direct effect on the mothers’ milk production. Furthermore, exposure to alcohol in mothers’ milk disrupted the infants’ sleep-wake pattern and motor development in ways that are contrary to the folklore. Based on these scientific studies, it would seem that the recommendation for a nursing mother to drink a glass of beer or wine shortly before nursing may actually be counterproductive, even though the mother may be more relaxed after a drink.

Scientific evidence such as that discussed above should not frighten women away from breastfeeding, however. It is not known how many women stop breastfeeding because of their concern about alcohol in their breast milk, thereby depriving their infants of the best nutrition available for them. Unlike the situation during pregnancy, when alcohol consumed at any time is passed on to the fetus, a lactating woman who drinks occasionally can limit her infant’s exposure to alcohol by not nursing for several hours after drinking, until the alcohol has been eliminated from her body and, consequently, her milk. Knowledge about the timing of alcohol’s transfer to the milk and about the potential effects that alcohol exposure via breast milk has on the infant is crucial for lactating women and health care professionals to make the best decisions for infants.

## Footnotes

1 Active sleep, also called rapid eye movement (REM) sleep, is the sleep stage during which dreaming occurs.
